# Fractional anisotropy and peripheral cytokine concentrations in outpatients with depressive episode: a diffusion tensor imaging observational study

**DOI:** 10.1038/s41598-022-22437-0

**Published:** 2022-10-19

**Authors:** Gebhard Sammer, Elena Neumann, Carlo Blecker, Bruno Pedraz-Petrozzi

**Affiliations:** 1 Psychiatry, Justus Liebig University Giessen, Klinikstrasse 36, 35392 Giessen, Hessen Germany; 2 Faculty of Psychology and Sports Science, Justus Liebig University Giessen, Giessen, Hessen Germany; 3 Bender Institute of Neuroimaging (BION), Faculty of Psychology and Sports Science, Justus Liebig University Giessen, Giessen, Hessen Germany; 4 Internal Medicine and Rheumatology, Campus Kerckhoff, Justus Liebig University Giessen, Giessen, Hessen Germany; 5 Central Institute of Mental Health, Mannheim, Germany

**Keywords:** Depression, Inflammation, Diffusion tensor imaging

## Abstract

Over the past few years, evidence of a positive relationship between inflammation and depression has grown steadily. The aim of the current study was to investigate whether such depression-related inflammation could also be associated with altered microstructural changes in the white matter. FA and serum cytokines (IL-1β, IL-6, TNF-α, and IFN-γ) were measured in 25 patients with depression (DE) and 24 healthy controls (HC). Diffusion tensor imaging was performed. Fractional anisotropy (FA) was calculated using the FSL pipeline for Tract-Based Spatial Statistics (TBSS). Both voxelwise and mean whole-brain FA were analyzed using general linear models (GLM). Higher concentrations of IL-1β were associated with lower whole-brain fractional anisotropy, particularly in people with depression (ρ = − 0.67; p < 0.001). TNF-α shared some variance with IL-1β and also showed a negative relationship between TNF-α concentrations and FA in depression (F_1,46_ = 11.13, p = 0.002, η^2^p = 0.21). In detail, the voxelwise analysis showed that the regression slopes of IL-1β on FA were more negative in the DE group than in the HC group, mainly in the corpus callosum (cluster statistics: genu corpus callosum, p = 0.022; splenium of corpus callosum, p = 0.047). Similar effects were not found for the other remaining cytokines. This study clearly demonstrated an association between peripherally measured IL-1β and white matter integrity in depression as assessed by DTI. The results suggest that microstructural changes in the corpus callosum are associated with increased peripheral IL-1β concentrations in depression.

## Introduction

Fractional anisotropy (FA) is a standard measurement in diffusion tensor imaging (DTI), which describes the movement of water molecules, from isotropic (FA = 0) to anisotropic movement (FA = 1). Examples of isotropic states are the cerebrospinal fluid (CSF) and the restriction of the movement of water molecules through membranes in fiber bundles for anisotropic states^1–3^.

A decrease in FA is associated with changes in white matter (WM), such as decreased axonal density and demyelination. In most cases, this correlates with a disease or a neuroinflammatory process^4–6^. Reduced FA levels have also been reported in different medical conditions, particularly mental disorders^7–9^, including depression^10^. In participants with depression, for instance, reduced FA values were observed for various WM fiber tissues^11–18^. Most of these studies included participants with first-episode depression^19–21^, acute exacerbation of depression^11,13^, drug-naïve patients^17–19^, and participants in inpatient or outpatient treatment^15,16,22–25^. Although an increasing body of literature supports reduced FA levels in depression, the pathophysiology of WM structural changes in depression is not clearly understood.

Likewise, alterations of the WM structure are observed when peripheral inflammation is present. Post-mortem and animal studies have demonstrated that peripheral inflammation is associated with WM injury^26,27^. Moreover, other experimental studies have also shown that psychological stress could cause the downregulation of the claudin-5, promoting the entrance of peripheral pro-inflammatory cytokines into the central nervous system and damaging the white matter integrity^28–30^. In addition, many studies have highlighted in the past decade the in vivo relationship between depression and peripheral inflammation^31–35^. Two meta-analyses by Köhler et al. and Dowlati et al*.* pointed out, for example, that patients with major depressive disorder (MDD) had increased peripheral pro-inflammatory cytokines such as IL-6 and TNF-α compared to healthy controls^31,36^. Another meta-analysis showed that IL-6 was increased in depressed subjects compared to healthy subjects^32^. A meta-analysis by *Howren *et al*.* showed positive correlations between depression and the cytokines IL-1 and IL-6 in patients with depression^37^. In addition, recent studies have shown significant correlations in depressive episodes between WM structures and peripheral inflammation^10,34,38^. In a volumetric MRI study by *Frodl *et al*.*, participants with depression who were not drug-naïve showed smaller hippocampal volumes but higher IL-6 concentrations^39^. Two further DTI studies showed negative correlations between the FA values of various WM fiber tracts and peripheral inflammatory cytokines (i.e. IL-1β and TNF-⍺), as well as with acute-phase inflammatory proteins (i.e. CRP) in patients with a first episode or drug-naïve depressed patients^28,34,38^.

This literature shows that peripheral inflammation and changes in WM microstructure are associated with depression^28,34,38^. It would be interesting to see this in drug-naïve first-episode depression and in people in outpatient treatment to define endophenotypes in depression^40^. Therefore, the main aim of this study was to investigate the relationship between WM integrity as measured by FA and peripheral inflammation in a community sample with and without depression. A negative association between peripheral inflammation and fractional anisotropy in depression is expected. However, no a priori assumptions were made about the regional distribution of these changes.

## Materials and methods

This work is part of a study on depression and also fatigue, which included the investigation with (sMRI, fMRI, DTI, EEG) in different samples. Results on cytokine concentrations, depressive symptoms and fatigue in the total sample of this project were published elsewhere by Pedraz-Petrozzi et al.^35^.

### Study participants

Fifty-one participants were recruited between June and September 2019. Two participants revoked their consent to have blood drawn while the experiment was being carried out and were therefore excluded from participation. In the end, one group consisted of 24 participants with a depressive episode (DE group) and another group of 25 participants without depression (healthy control group or HC group). The groups were frequency-matched according to age and gender. The main characteristics of the DE and HC groups are reported in Table 1. Participants in the DE group had received medical, psychotherapeutic, or both types of treatment for at least 6 months before this study. As inclusion criterion, participants of the DE group had to meet the ICD-10 criteria (World Health Organization, 10th version of the International Classification of Diseases) for a depressive episode. The rating was carried out by clinical experts from the Department of Psychiatry at the University Hospital of Giessen^35,41^. People with psychotic disorders, current psychotic episodes, or any medical condition were not included in the study; however, participants with a comorbid psychiatric disorder, such as personality or anxiety disorders, were included if they had a predominant depressive episode in the past 6 months. Individuals could not participate if they suffered from acute or chronic disease or illness, particularly infection-related, except depression.

**Table 1:** General and laboratorial characteristics of the DE and HC groups.

	DE (n = 24)	HC (n = 25)	p value
Age (in years)	31.88 (12.76)	26.80 (8.72)	0.113
BMI (in kg/m^2^)	24.05 (4.16)	24.86 (5.86)	0.581
Female:male	15:9	15:10	0.858
Psychiatric medication (y:n)	19:5	0:25	< 0.001
Smoking behavior (y:n)	3:21	2:23	0.667
Disease duration (in years)	9.16 (7.84)		
BDI-FS	7.25 (5.17)	1.80 (2.16)	< 0.001
**Cytokines (in pg/mL)**			
IL-6	2.76 (2.09)	1.72 (1.33)	0.045
TNF-α	8.81 (1.40)	9.12 (1.17)	0.417
IL-1β	1.02 (0.68)	1.19 (0.98)	0.483
IFN-γ	17.43 (10.64)	15.95 (3.11)	0.516

*BMI* body mass index, *BDI-FS* Beck depression inventory fast screening, *IL-6* Interleukin 6, *TNF-α* tumor necrosis factor alpha, *IL-1β* interleukin 1 beta, *IFN-γ* interferon gamma, *DE* depression group, *HC* healthy controls. Continuous variables are expressed as mean (standard deviation).

No participant in the HC group was taking any psychotropic drugs. In the group of depressed participants (DE), 18 of 24 received pharmacotherapy, 14 of whom received monotherapy with antidepressants (4 with escitalopram, 1 with sertraline, 2 with citalopram, 1 with paroxetine, 2 with fluoxetine, 1 with opipramol, 1 with duloxetine, and 1 with venlafaxine) or quetiapine (1 participant). Another 3 participants were treated with 2 antidepressants (sertraline + amitriptyline, venlafaxine + mirtazapine, and citalopram + bupropion), and one participant was treated with a combination of duloxetine and prothipendyl. Finally, 1 participant received diazepam *pro re nata* (PRN). The remaining participants indicated that they had been without pharmacological treatment for at least 6 months.

Insufficient knowledge of German and severe somatic restrictions, such as severe visual and hearing impairments, or age over 65 years, were considered as exclusion criteria. Other exclusion criteria corresponding to the safety instructions of the MRI scanner manufacturer were also considered in this study.

The estimated power of the total sample was estimated using G*Power 3.1 (N = 49, power = 0.95) and is above the accepted minimum power threshold (1 − β = 0.80), which indicates that the sample size for this study design is sufficient to meet the study objectives to reach.

### Evaluation of depression symptoms: BDI-FS

The German validated version of the Beck Depression Inventory—*fast screening* (BDI-FS) was used to assess the symptom burden of the depression for each participant^35,42^. The BDI-FS is a validated short version of the BDI-II, which evaluates self-criticism, self-aversion, past failure, pessimism, anhedonia, sadness, and suicidal behavior (i.e., thoughts or wishes). The BDI-FS consists of 7 items, and the scores ranged between 0 and 21 points. A higher score reflects a higher symptom burden of a depressive episode. The scores can be summarized in four different categories (*minimal*, *mild*, *moderate*, and *severe*) based on the total number of points (*minimal*: 0–3 points; *mild*: 4–8 points; *moderate*: 9–12 points; *severe*: 13–21 points)^35^.

### Peripheral inflammatory markers—cytokines

Between 8:00 am and 12:00 pm, peripheral venous blood samples were collected with potassium Ethylenediaminetetraacetic acid sample tubes (K-EDTA, SARSTEDT AG & Co. KG, Nümbrecht, Germany). Blood samples were recollected after 12 h of fasting. After recollection, the blood samples were centrifuged for 15 min at 4 °C at 1100×*g*, and the collected plasma was immediately stored at – 20 °C. The plasma tubes were delivered to the clinical immunology research facilities of the Justus Liebig University Giessen, within a maximum of 4 weeks and stored at – 80 °C. Four different cytokines (interleukin 6 or IL-6, interleukin 1 beta or IL-1β, tumor necrosis factor alpha or TNF-α, and interferon-gamma or IFN-γ) were measured using Quantikine ELISA kits (R&D Systems Inc., Minneapolis, Minnesota, United States of America). Intra- and inter-precision values were < 10%. Any values below the minimum detectable dose were considered zero and included in the analysis. The selected cytokines’ concentration was estimated using Tecan Reader with the Magellan Reader Software (Tecan Group Ltd., Männedorf, Switzerland). Parameters were calculated using Marquardt’s 4-parameter estimation method.

### Diffusion tensor imaging

#### Data acquisition

All participants were subjected to a DTI imaging protocol with a SIEMENS MAGNETROM Prisma 3.0 Tesla MRI scanner and a 64-channel head coil (Bender Institute for Neuroimaging, Faculty of Psychology, Justus-Liebig University Giessen).

The DTI protocol consisted of two measurements, anterior–posterior (AP) and posterior–anterior (PA). The AP DTI image protocol was measured with the following settings: TR/TE = 6200 ms/67 ms, 60 slices, slice thickness = 2 mm, field of view (FoV) = 232 mm, number of excitations = 1, and spatial resolution = 2 × 2 × 2 mm, diffusion gradient (b value) = 2000 s/mm^2^, duration = 7 min and 40 s. Regarding the diffusion gradient, an MDDW diffusion mode (Siemens Multidirectional Diffusion Imaging) with 64 directions and 2 weightings was used. The PA DTI image protocol was used with settings similar to the AP protocol: TR/TE = 6200 ms/67 ms, 60 slices, slice thickness = 2 mm, field of view (FoV) = 232 mm, number of excitations = 1 and spatial resolution = 2 × 2 × 2 mm, diffusion gradient (b-value) = 2000 s/mm^2^, duration = 1 min and 41 s. Similar diffusion gradient settings were used in the PA imaging protocol, namely an MDDW diffusion mode with 6 directions and 2 weightings.

#### Image processing

Fractional anisotropy (FA) was chosen as the preferred measure in this study because it is the most summary measure of microstructural integrity, which has been extensively shown to be very sensitive to different types of microstructural changes. However, it should be noted that other DTI measurements, including axial or radial diffusion or free water analysis, have recently been used in inflammation and MDD research and may represent promising approaches^43,44^.

FA was computed using the FMRIB Software Library (FSL)^45^ FDT-processing pipeline. No settings had to be adjusted. Dicom data were converted into NIFTY, vector orientation was checked, susceptibility-induced distortion correction (fieldmap estimation) was performed using topup^46^, brain extraction applied BET^47^ on the output of topup. Distortion correction (eddy currents, susceptibility-induced distortions, and subject’s motion) was conducted using eddy^48^ with a fieldmap estimated by topup. Further, diffusion tensors were fitted on the eddy-corrected data using *dtifit*. For voxel-wise statistical analyses, data were processed applying the standard FSL pipeline for TBSS^49^. Finally, the whole-brain FA was calculated as the average of all non-zero values of each FA-skeleton from the participants.

#### Statistical analysis

General information, including clinical data and peripheral cytokine concentrations, are displayed as tables. Continuous variables are presented using measures of central tendency (mean, standard deviation) and dichotomous categorical variables using frequency and count data. T-tests (continuous variables) or Fisher’s exact tests (dichotomous categorical variables) were applied for differences between groups. The means were considered different if they had a two-sided p value ≤ 0.05. In this case, homogeneity between the groups was assumed if the p value was greater than 0.05.

To evaluate the relationship between WM integrity and peripheral inflammation, correlations between the mean whole-brain FA values, the four cytokines, and the BDI-FS were tested first for each group separately, following the recommendations of previous studies^50,51^. Furthermore, the correlations were partialized and controlled for *age* and *BMI* since these variables are frequently confounding factors in studies involving FA values^4,52^ and cytokines^53,54^. Regarding the multiple testing issue, the p values were adjusted following the number of pro-inflammatory markers and BDI-FS scores, defining significance finally for these correlations as p_BONF_ = 0.01 and presenting these results in Table 2. The obtained correlation matrix also gives relevant information for the voxelwise analysis procedure described below.

**Table 2:** Spearman rank correlations between FA (whole brain), BDI-FS values, IL-6, TNF-ɑ, IL-1β, and IFN-ɣ.

	FA (whole-brain)	BDI-FS	IL-6	TNF-ɑ	IL-1β	IFN-ɣ
**FA (whole-brain)**						
HC	–					
DE						
**BDI-FS**						
HC	0.346	–				
DE	0.254					
**IL-6**						
HC	0.092	**0.622	–			
DE	− 0.178	0.128				
**TNF-ɑ**						
HC	0.239	0.142	0.122	–		
DE	*− 0.526	0.264	0.057			
**IL-1β**						
HC	0.014	− 0.064	− 0.182	− 0.016	–	
DE	***− **0.666**	− 0.067	0.363	**0.557		
**IFN-ɣ**						
HC	0.171	0.223	0.157	**0.605	− 0.321	–
DE	− 0.172	0.216	0.068	0.333	0.234	

Correlations are controlled for the variables age and BMI. p values are marked as following: *p < 0.05, **p < 0.01, ***p < 0.001. Moreover, p values lesser or equal than the Bonferroni adjusted threshold (P_BONF_ = 0.01) are marked in bold.

**Figure 1:**
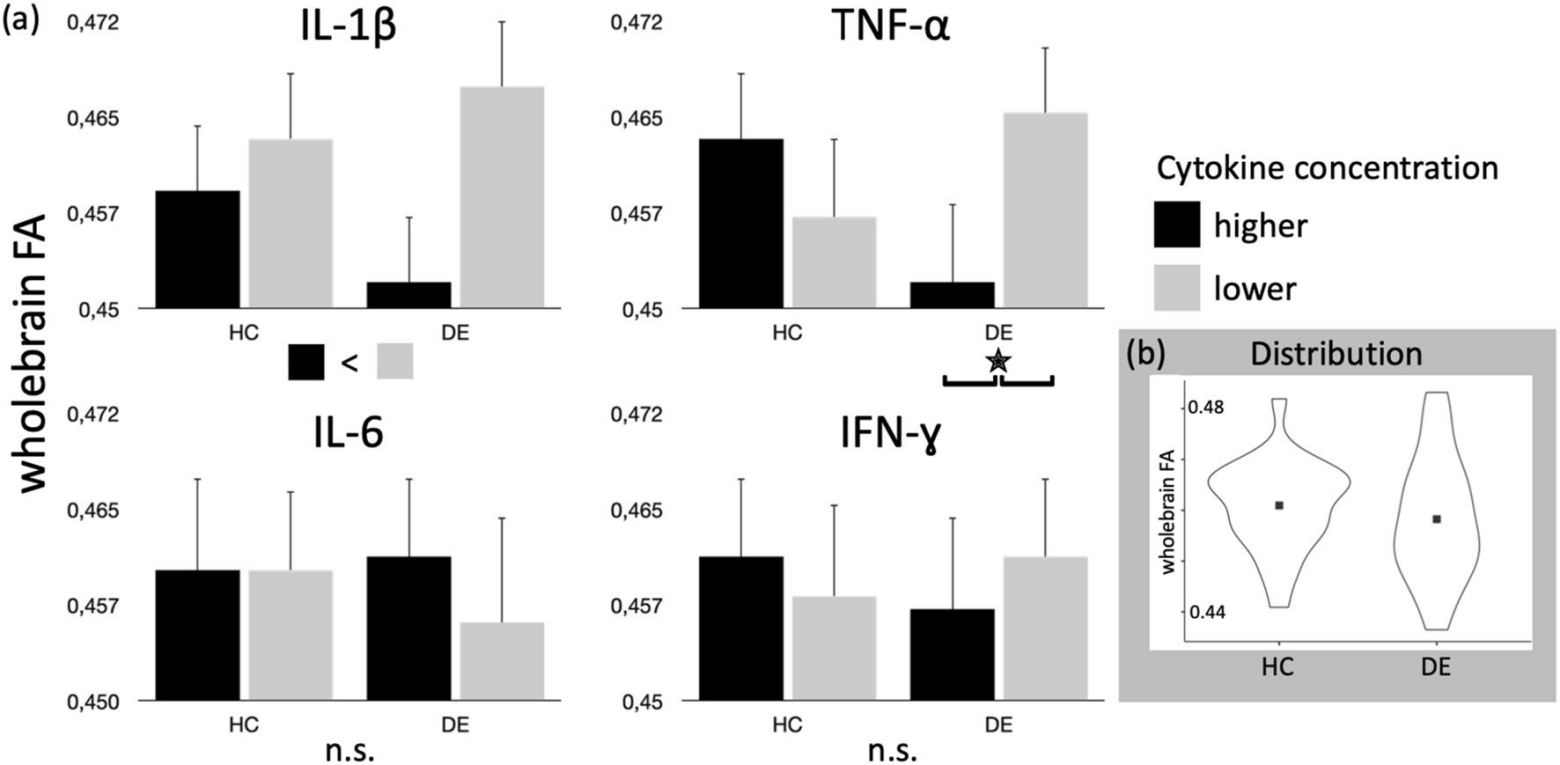
(**a**) Results of the GLM on the whole-brain FA with factors group status (HC; DE) and dichotomized cytokine concentration (median split: lower; higher). Age was included as a covariate. The interaction plots of group x cytokine concentration are displayed. Bar height represents the mean FA values, error bars indicate the CI95+. IL-1β showed a main effect (p = 0.002) but no interaction effect (p = 0.061). TNF-ɑ showed no main effect (p = 0.286), but an interaction effect (p = 0.002). Both effects survive Bonferroni adjusted threshold of p_BONF_ = 0.0125. IL-6 and IFN-ɣ showed no effects over threshold. (**b**) Violin plots representing the distribution of whole-brain FA within groups HC and DE.

In addition, differences between BDI-FS scores and pro-inflammatory cytokines on the mean whole-brain FA were analyzed using a general linear model (GLM) with ANOVA omnibus tests. This model consisted of the factors *group status* (i.e., DE and HC group) and the dichotomized cytokine concentrations using the median split method. Other variables, such as *age* and BDI-FS scores, were included in this model as covariates. A separate ANOVA was conducted for each cytokine, and the resulting p values were adjusted using Bonferroni correction for multiple tests (p_BONF_ = 0.05/4 = 0.0125). Moreover, effect sizes were calculated using partial eta-squared (η^2^_p_) values. The effects for both partial ES formulae were defined as following: *very small* (η^2^_p_ < 0.01), *small* (0.01 ≤ η^2^_p_ < 0.06), *moderate* (0.06 ≤ η^2^_p_ < 0.14) and *large* (η^2^_p_ ≥ 0.14)^55,56^. The results of this model are presented in Fig. 1.

Both correlation and analysis of variance were performed using SPSS version 26.0 (International Business Machines Corporation, New York, United States of America) or jamovi 2.0.0^57^ together with the toolbox GAMLj^58^.

**Figure 2:**
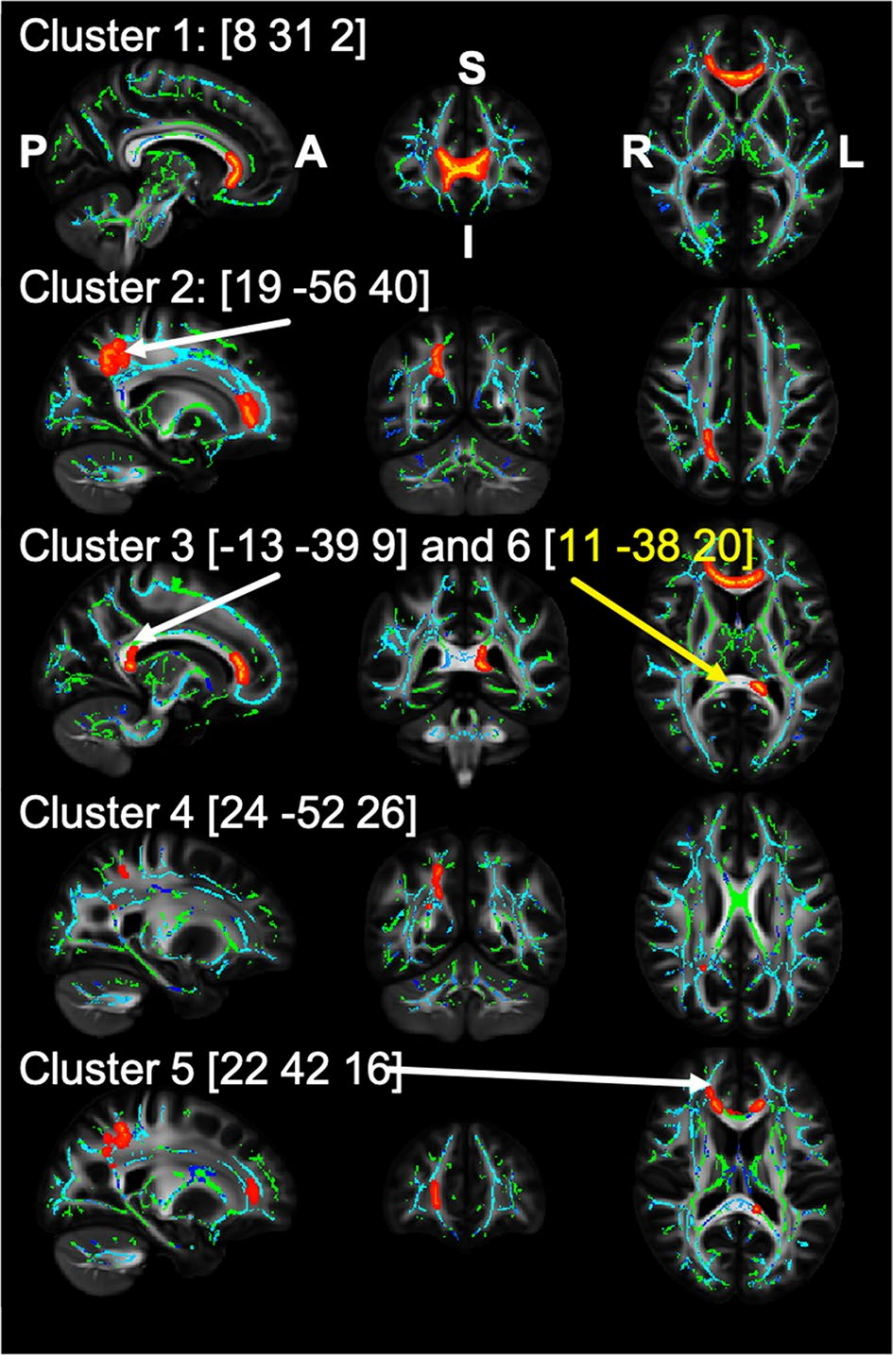
FA results for the interaction effect of group status (DE, HC) * IL-1β. Each row shows the sagittal, coronal, and axial views aligned with the coordinates of the respective cluster maximum (radiological display convention). Each slice consists of an FA template (grey), the mean FA skeleton of this study (green), overlaid by the age effect (blue; p_FWE_ ≤ 0.05) and by the thickened threshold statistic with p_FWE_ ≤ 0.05 (red-yellow), representing more positive slopes in the HC group. The clusters refer to the descriptions in Table 3: Cluster 1—Genu Corpus Callosum; Cluster 2—Superior longitudinal fasciculus L; Cluster 3 & 6—Splenium Corpus Callosum; Cluster 4—Posterior Corona Radiata R; Cluster 5—Anterior Corona Radiata R. *R/L* right/left hemisphere, *P/A* posterior/anterior, *I/S* inferior/superior.

Finally, voxel-wise statistical analyses were performed using a GLM to determine possible relationships with inflammatory parameters in particular WM structures. The design matrix consisted of regressors for the *group status*, *age*, *gender*, *body mass index* (BMI in kg/m^2^), *BDI-FS values*, and the within-covariates for the peripheral cytokine concentrations. This model estimation was performed using the FSL randomize tool using a sample of 5000 permutations combined with the Threshold-Free Cluster Enhancement^59^. Contrast calculation included the mean group differences, the effects of the covariates, and the group differences of the slope of the regression of FA on the peripheral cytokines. In addition, F-contrasts were calculated to examine the effect of the combination of all cytokines. Result maps are presented in Fig. 2 and thresholded at p_FWE_ ≤ 0.05 (corrected for multiple comparisons across space).

### Ethical approval and admission to participation

Each participant or its legal authorized representative was fully informed of this study and gave their written consent to participate. This study was approved by the ethics committee of the Medical Faculty of the JLU (file number AZ 81/18) and carried out in accordance with the Helsinki Declaration and the ethical standards of the APA. This study is part of a project to investigate inflammatory factors and fatigue in patients with depression and multiple sclerosis. Declarations of consent in the original language (German) are available on request.

## Results

### Sample description

Regarding *age* (t = − 1.62, p = 0.113), *gender* (χ^2^ = 0.03, df = 1, p = 0.858), *smoking behavior* (χ^2^ = 0.27, df = 1, p = 0.667) and *BMI* (t = 0.56, p = 0.581) no differences were found between DE and HC. However, the BDI-FS values were higher in DE than in HC (t = − 4.78, p < 0.001). The IL-6 concentrations were higher in DE compared to HC (t = − 2.07, p = 0.045), no differences between the two groups were found with regard to the remaining peripheral cytokines (Table 1).

### Fractional anisotropy (FA)

#### Mean whole-brain FA analysis

Whole-brain FA correlations with cytokine correlation and BDI-FS values within each group are shown in Table 2. This analysis found significant correlations for FA values with IL-1β and TNF-ɑ in the DE group. However, only IL-1β survived the Bonferroni-adjusted threshold. Both correlations were negatively signed, indicating less FA with higher cytokine concentration.

The GLM analysis revealed a main effect of IL-1β on the whole-brain FA (F_1,46_ = 10.40, p = 0.002, η^2^p = 0.21), showing lower FA in the subgroup having higher IL-1β concentration (Fig. 1). Similar effects were not seen for the other cytokines: IL-6 (F_1,46_ = 0.41, p = 0.525, η^2^p = 0.01), TNF-ɑ (F_1,46_ = 1.17, p = 0.286, η^2^p = 0.03), IFN-γ (F_1,46_ = 0.00, p = 0.98, η^2^p = 0.00). However, TNF-ɑ concentrations showed an interaction effect with *group status*, showing only differences in the DE group (F_1,46_ = 11.13, p = 0.002, η^2^p = 0.21). Once more, higher cytokine concentrations were associated with lower FA. Both results also survive the Bonferroni adjusted threshold. The results of this analysis are summarized in Fig. 1.

#### Voxelwise FA analysis

Voxelwise FA analysis did not show effects of *group*, *sex*, *BMI*, or *BDI-FS values*. However, the covariable *age* explained a substantial portion of variance (Fig. 2). Comparing the slopes of the regression of FA on the peripheral cytokines, a significant effect was found in the analysis including the IL-1β concentrations (Fig. 2). Here, the slope for the HC group was significantly more positive than the slope in the DE group. The detailed statistics, including t and p values for the clusters and voxel-by-voxel statistical parametric threshold maps, are presented in Fig. 2 and Table 3. Concerning the presented results, three clusters cover the genu of corpus callosum and splenium of the corpus callosum. The remaining three clusters contain the superior longitudinal fasciculus, the anterior and the posterior corona radiata. A similar effect as described for IL-1β was not found for the other three cytokines (IL-6, TNF-α and IFN-γ). A combination of the contrasts for all four cytokines, as can be tested using F-contrasts, did not show an effect above the threshold.

**Table 3:** Voxel-wise analysis of the interaction effect Group *IL-1β.

Cluster	Anatomical region	Cluster size	p_FWE_	t-value (max)	MNI coordinates (mm)		
					x	y	z
1	Genu of corpus callosun^*#*^	1083	0.022	5.45	8	30	1
2	Superior longitudinal fasciculus^§,+^ L (> 90%)	324	0.043	3.96	19	− 57	39
3	Splenium of corpus callosum^*#*^	75	0.047	4.38	− 13	− 40	8
4	Posterior Corona Radiata^#^, R	4	0.05	3.26	24	− 53	25
5	Anterior Corona Radiata^#^, R	1	0.05	2.24	21	44	8
6	Splenium of corpus callosum^*#*^	1	0.05	4.56	10	− 38	20

^#^JHU ICBM-DTI-81 White Matter Atlas.

^§^Not classified in JHU ICBM-DTI-81 White-Matter atlas.

^+^XTRACT HCP Probabilistic Tract Atlases.

Clusters with p_FWE_ ≤ 0.05 are displayed. *L* left hemisphere, *R* right hemisphere, *MNI* Montreal Neurologic Institute, *p*_*FWE*_ family-wise error corrected p value.

## Discussion

The main result of this study showed an altered association of IL-1β with fractional anisotropy, as can be measured using diffusion tensor imaging, in depression. The most intriguing fact is thereby, that this result was not found in the comparison of healthy with severely depressed or unmedicated first episode people^34^ but it was found in a community sample where the people suffering from depression were outpatients mostly under pharmacological treatment. The “healthy controls” were also part of a community sample that reported fewer symptoms of depression. It was shown that the IL-1β concentration correlated negatively with the wholebrain FA in the group with depression. Correlation of age and BMI with FA was always taken into account. In the group without depression this relationship did not occur. That means that in people with depression FA values were less when the cytokine concentration was higher. Whether the participants had a depression or not, in average the FA was less with higher IL-1β cytokine concentration.

Concretizing the localisatory aspect of this result, the voxelwise FA analysis showed that that the slopes of the regression of FA on IL-1β was different in the groups. That means that the IL-1β concentration correlated more negatively in people with depression than without depression. This effect predominantly occurred in the big commissural fibers of the brain, the genu and splenium of the corpus callosum. The same effects occurred also in the superior longitudinal fasciculus, the anterior and the posterior corona radiata. Similar effects could not be shown for the other three cytokines. A basic association between microstructural changes (lower FA) and major depression has been reported for all of these structures^34,60,61^.

Many studies have shown that IL-1β in particular has a negative impact on myelination and WM microstructure and that its pharmacological blockade could reduce apoptosis, microgliosis, and inflammation^27,62–64^. Those changes have also been found in animal models for depression^65,66^. It is therefore to be expected that FA and IL-1β correlate more negatively in people with depression than without depression. Likewise, human studies have shown negative correlations between markers of peripheral inflammation and mean whole brain FA in other diseases with a neuroinflammatory component^67,68^. With regard to depression, only one human study reported a similar result with regard to peripheral IL-1β and WM integrity^34^ and showed between-group effects of IL-1β levels in the inferior fronto-occipital fasciculus (IFOF) and in the genu of the corpus callosum (GCC) first episode of treatment-naïve depression. This was partly shown in the current study with depressed outpatients. An interaction effect of the group * covariate of the IL-1β level was found in the GCC, but not in the inferior fronto-occipital fasciculus (Fig. 2). Instead, an effect was found in the superior longitudinal fasciculus, the anterior and the posterior corona radiata. However, due to inconsistencies, the published coordinates are not always easy to assess, so overlaps cannot be ruled out. Since both studies showed a similar interaction effect in samples with different properties, first episode treatment-naïve^34^ vs. non-drug-naïve treated outpatients, at least the correlation between corpus callosum and peripheral inflammation may be a characteristic of depression regardless of treatment.

Higher TNF-α levels were also associated with lower whole-brain FA levels in the depressed group when groups with higher and lower cytokine concentrations were compared. In the voxelwise analysis, however, this cytokine with p_FWEmax_ = 0.088 just missed the voxel threshold of p_FWE_ = 0.05 and was therefore not considered relevant for the interpretation. A DTI-based meta-analysis suggested the influence of inflammatory cytokines (i.e., IL-1β, IL-6, and TNF-α) on changes in the microstructural integrity of the WM^10^. However, this could only be shown, probably due to variance issues, when the TNF-α distribution was dichotomized by a median split, although TNF-α and IL-1β shared 31% of the variance in the group with depression. Another probable reason might be group-related differences between depression participants. There is only one study with the voxelwise approach that showed a connection between TNF-α and FA in depression^38^, which, however, differs from the results of the current study and the study cited above^34^. To speculate, individual differences could account for the different outcomes, i.e., macrophages, for example, produce cytokines, IL-1β and TNF-α, which have an inflammatory effect^69^. Nevertheless, in some groups with depression one cytokine predominates over the other^34,38^. That could be an interesting starting point for further studies.

The influence of the medication in the depressive group on the results is difficult to classify. There are some recent studies that make the problem clear. In drug-naïve MDD patients, higher levels of FA were found in some white matter structures but not in the corpus callosum after treatment with venlafaxine plus benzodiazepines PRN^70^. IL-1ß effects were not observed, but an inverse correlation between the left posterior limb of the inner capsule and the highly sensitive C-reactive protein was reported, regardless of treatment. Another recent study examined the relationship between the IL-8/IL-10 ratio and free water before and after treatment with ketamine^44^. The antidepressant medication of the patient group was very similar to the current study. The results were not comparable to the current study. Ketamine infusion had no effect on WM microstructure but reduced the IL-8/IL-10 ratio. In a combined DTI and immunological study of bipolar disorder, including a sample of depressed participants, effects occurred in bipolar patients and no IL-1β effects were reported^71^. Sugimoto et al., studying drug-naïve MDD patients, found an association between IL-1ß levels and the genu corpus callosum^34^, as did the current study of participants taking antidepressants. Lim al. found higher TNF-α and IL-8 and lower FA in the corpus callosum, anterior corona radiate, superior corona radiate, and superior longitudinal fasciculus in untreated but not drug-naïve participants with MDD compared to an HC group. IL-1β levels were quite lower than in our study, other cytokine levels were equal to or lower than in our study^38^. Although there was no correlation with IL-1β, the highlighted MW structures are very similar to our study. The clusters we found are smaller, the sample size was about the same, as was the difference in depressiveness between groups. Given the results of these more recent studies, it seems unlikely that an association of IL-1β and FA in the corpus callosum, the superior longitudinal fasciculus, and the anterior corona radiata is primarily due to drug effects. In addition to medication, the influence of psychotherapy on the hypothalamic–pituitary–adrenal axis (HPA) and immune regulation and their possible connection should not be neglected. In participants with depression, Doolin et al. found a negative association between IL-1β, mRNA and morning cortisol^72^. Effects of non-drug therapies or treatments such as psychotherapy or even exercise, both of which have at least low evidence effects on the HPA axis and immune regulation, were not considered in any of the cited studies and would be an important topic for future depression research.

The strength of this study was to examine a sample of outpatients diagnosed with a depressive episode, who were medicated and who were not first-episode patients, showing an effect of the proinflammatory cytokine IL-1β on DTI measures. This allows the findings to be extended to a broader population of people with depression. Nevertheless, some limitations should be considered. (1) IL-1β concentrations were close to the lowest limit of detection, as shown in Table 1. The work of *Sugimoto *et al*.*^34^, which shared similarities with this study, also reported low IL-1β concentrations near the lowest limit of detection. (2) The results of this study cannot say anything about causality, of whether depression triggers inflammation or vice versa. (3) The heterogeneity of the depressed patients represented a limitation in this study, since participants with comorbidity (personality and anxiety disorders) were included in this study. However, these participants reported a predominant depressive episode in the last 6 months and thus met the inclusion requirements. (4) In our view, it is not a problem that there were more female participants than male participants, as this ratio is consistent with statistical and epidemiological data on depression worldwide.

In conclusion, proinflammatory IL-1β and TNF-α showed to be more negatively correlated with white matter FA of the brain in outpatients with depression, particularly for IL-1β in the corpus callosum. Future studies should consider further examining the correlations between IL-1β and FA levels of WM structures at different stages of depressive disorder (e.g., healthy controls, treated depressed patients, and untreated first-episode depressed patients) or in different types of depression (e.g., atypical depression, depression with somatic symptoms, bipolar vs. unipolar depression). Longitudinal methods could help to decipher the time course of inflammation, microstructural changes and depression. Finally, fractional anisotropy (FA) as a summary measure of microstructural integrity, which has been extensively shown to be very sensitive to different types of microstructural changes, seemed to be an appropriate measure in the context of this study. However, it is recommended to include other DTI measures such as axial and radial diffusion or free water others to learn about the nature of the change.

## Data Availability

The data that support the results of this study are not publicly available due to the applicable national data protection law, but can be requested from the respective author upon justified request.

## References

[1] Grieve SM, Williams LM, Paul RH, Clark CR, Gordon E (2007). Cognitive aging, executive function, and fractional anisotropy: a diffusion tensor MR imaging study. Am. J. Neuroradiol..

[2] Alba-Ferrara LM, de Erausquin GA (2013). What does anisotropy measure? Insights from increased and decreased anisotropy in selective fiber tracts in schizophrenia. Front. Integr. Neurosci..

[3] Minati L, Węglarz WP (2007). Physical foundations, models, and methods of diffusion magnetic resonance imaging of the brain: A review. Concepts Magn. Reson. Part A.

[4] Bettcher BM (2013). Body mass and white matter integrity: The influence of vascular and inflammatory markers. PLoS One.

[5] Bettcher BM (2015). Declines in inflammation predict greater white matter microstructure in older adults. Neurobiol. Aging.

[6] Wersching H (2010). Serum C-reactive protein is linked to cerebral microstructural integrity and cognitive function. Neurology.

[7] Kelly S (2018). Widespread white matter microstructural differences in schizophrenia across 4322 individuals: results from the ENIGMA Schizophrenia DTI Working Group. Mol. Psychiatry.

[8] Phan KL (2009). Preliminary evidence of white matter abnormality in the uncinate fasciculus in generalized social anxiety disorder. Biol. Psychiatry.

[9] Versace A (2008). Elevated left and reduced right orbitomedial prefrontal fractional anisotropy in adults with bipolar disorder revealed by tract-based spatial statistics. Arch. Gen. Psychiatry.

[10] Murphy ML, Frodl T (2011). Meta-analysis of diffusion tensor imaging studies shows altered fractional anisotropy occurring in distinct brain areas in association with depression. Biol. Mood Anxiety Disord..

[11] Hermesdorf M (2017). Reduced fractional anisotropy in patients with major depressive disorder and associations with vascular stiffness. NeuroImage Clin..

[12] Carballedo A (2012). Reduced fractional anisotropy in the uncinate fasciculus in patients with major depression carrying the met-allele of the Val66Met brain-derived neurotrophic factor genotype. Am. J. Med. Genet. B Neuropsychiatr. Genet..

[13] Repple J (2020). Severity of current depression and remission status are associated with structural connectome alterations in major depressive disorder. Mol. Psychiatry.

[14] Coloigner J (2019). White matter abnormalities in depression: A categorical and phenotypic diffusion MRI study. NeuroImage Clin..

[15] Osoba A (2013). Disease severity is correlated to tract specific changes of fractional anisotropy in MD and CM thalamus—a DTI study in major depressive disorder. J. Affect. Disord..

[16] Poletti S (2018). Impact of early and recent stress on white matter microstructure in major depressive disorder. J. Affect. Disord..

[17] Won E (2016). Association between reduced white matter integrity in the corpus callosum and serotonin transporter gene DNA methylation in medication-naive patients with major depressive disorder. Transl. Psychiatry.

[18] Liu X (2016). Relationship between white matter integrity and serum cortisol levels in drug-naive patients with major depressive disorder: Diffusion tensor imaging study using tract-based spatial statistics. Br. J. Psychiatry.

[19] Guo W (2012). Altered white matter integrity in young adults with first-episode, treatment-naive, and treatment-responsive depression. Neurosci. Lett..

[20] Ma N (2007). White matter abnormalities in first-episode, treatment-naive young adults with major depressive disorder. Am. J. Psychiatry.

[21] Yuan Y (2007). White matter integrity of the whole brain is disrupted in first-episode remitted geriatric depression. NeuroReport.

[22] Taylor WD (2008). Frontal white matter anisotropy and antidepressant remission in late-life depression. PLoS One.

[23] Wang T (2013). Early-stage psychotherapy produces elevated frontal white matter integrity in adult major depressive disorder. PLoS ONE.

[24] Yang X (2017). White matter microstructural abnormalities and their association with anticipatory anhedonia in depression. Psychiatry Res. Neuroimaging.

[25] Pfarr J (2021). Brain structural connectivity, anhedonia, and phenotypes of major depressive disorder: A structural equation model approach. Hum. Brain Mapp..

[26] Boroujeni ME (2021). Inflammatory response leads to neuronal death in human post-mortem cerebral cortex in patients with COVID-19. ACS Chem. Neurosci..

[27] Kelly SB (2021). Interleukin-1 blockade attenuates white matter inflammation and oligodendrocyte loss after progressive systemic lipopolysaccharide exposure in near-term fetal sheep. J. Neuroinflamm..

[28] Thomas M (2021). Elevated systemic inflammation is associated with reduced corticolimbic white matter integrity in depression. Life.

[29] Menard C (2017). Social stress induces neurovascular pathology promoting depression. Nat. Neurosci..

[30] Dudek KA (2020). Molecular adaptations of the blood–brain barrier promote stress resilience vs depression. Proc. Natl. Acad. Sci..

[31] Köhler CA (2017). Peripheral cytokine and chemokine alterations in depression: a meta-analysis of 82 studies. Acta Psychiatr. Scand..

[32] Hiles SA, Baker AL, de Malmanche T, Attia J (2012). A meta-analysis of differences in IL-6 and IL-10 between people with and without depression: Exploring the causes of heterogeneity. Brain. Behav. Immun..

[33] Maes M, Mihaylova I, Kubera M, Ringel K (2012). Activation of cell-mediated immunity in depression: Association with inflammation, melancholia, clinical staging and the fatigue and somatic symptom cluster of depression. Prog. Neuropsychopharmacol. Biol. Psychiatry.

[34] Sugimoto K (2018). Relationship between white matter integrity and serum inflammatory cytokine levels in drug-naive patients with major depressive disorder: diffusion tensor imaging study using tract-based spatial statistics. Transl. Psychiatry.

[35] Pedraz-Petrozzi B, Neumann E, Sammer G (2020). Pro-inflammatory markers and fatigue in patients with depression: A case–control study. Sci. Rep..

[36] Dowlati Y (2010). A meta-analysis of cytokines in major depression. Biol. Psychiatry.

[37] Howren MB, Lamkin DM, Suls J (2009). Associations of depression with C-reactive protein, IL-1, and IL-6: A meta-analysis. Psychosom. Med..

[38] Lim J, Sohn H, Kwon M-S, Kim B (2021). White matter alterations associated with pro-inflammatory cytokines in patients with major depressive disorder. Clin. Psychopharmacol. Neurosci..

[39] Frodl T (2012). Reduced expression of glucocorticoid-inducible genes GILZ and SGK-1: high IL-6 levels are associated with reduced hippocampal volumes in major depressive disorder. Transl. Psychiatry.

[40] Zobel A, Maier W (2004). Endophaenotypen—ein neues Konzept zur biologischen Charakterisierung psychischer Stoerungen. Nervenarzt.

[41] World Health Organization. *The ICD-10 Classification of Mental and Behavioural Disorders: Diagnostic Criteria for Research* (1993).

[42] Kliem S, Mößle T, Zenger M, Brähler E (2014). Reliability and validity of the beck depression inventory-fast screen for medical patients in the general German population. J. Affect. Disord..

[43] Bergamino M, Pasternak O, Farmer M, Shenton ME, Paul Hamilton J (2016). Applying a free-water correction to diffusion imaging data uncovers stress-related neural pathology in depression. NeuroImage Clin..

[44] Langhein M (2022). Association between peripheral inflammation and free-water imaging in Major Depressive Disorder before and after ketamine treatment—a pilot study. J. Affect. Disord..

[45] Jenkinson M, Beckmann CF, Behrens TEJ, Woolrich MW, Smith SM (2012). FSL. Neuroimage.

[46] Andersson JLR, Skare S, Ashburner J (2003). How to correct susceptibility distortions in spin-echo echo-planar images: application to diffusion tensor imaging. Neuroimage.

[47] Smith SM (2002). Fast robust automated brain extraction. Hum. Brain Mapp..

[48] Andersson JLR, Sotiropoulos SN (2016). An integrated approach to correction for off-resonance effects and subject movement in diffusion MR imaging. Neuroimage.

[49] Smith SM (2006). Tract-based spatial statistics: Voxelwise analysis of multi-subject diffusion data. Neuroimage.

[50] Marzban, C., Illian, P. R., Morison, D. & Mourad, P. D. Within-group and between-group correlation: Illustration on noninvasive estimation of intracranial pressure (2013).

[51] Ranganathan P, Pramesh C, Aggarwal R (2017). Common pitfalls in statistical analysis: Logistic regression. Perspect. Clin. Res..

[52] Nenonen M (2015). Possible confounding factors on cerebral diffusion tensor imaging measurements. Acta Radiol. Open.

[53] Azizian M (2016). Cytokine profiles in overweight and obese subjects and normal weight individuals matched for age and gender. Ann. Clin. Biochem..

[54] El-Mikkawy DME, El-Sadek MA, El-Badawy MA, Samaha D (2020). Circulating level of interleukin-6 in relation to body mass indices and lipid profile in Egyptian adults with overweight and obesity. Egypt. Rheumatol. Rehabil..

[55] Morris PE, Fritz CO (2013). Effect sizes in memory research. Memory.

[56] Cambridge, U. of. Rules of thumb on magnitudes of effect sizes. *2019* http://imaging.mrc-cbu.cam.ac.uk/statswiki/FAQ/effectSize.

[57] Love, J. *et al.* The jamovi project. https://www.jamovi.org (2020).

[58] Gallucci, M. GAMLJ—General Analyses for Linear Models. https://www.jamovi.org/library.html (2019).

[59] Smith S, Nichols T (2009). Threshold-free cluster enhancement: Addressing problems of smoothing, threshold dependence and localisation in cluster inference. Neuroimage.

[60] Pisner DA, Shumake J, Beevers CG, Schnyer DM (2019). The superior longitudinal fasciculus and its functional triple-network mechanisms in brooding. NeuroImage Clin..

[61] Choi S (2015). Association of brain-derived neurotrophic factor DNA methylation and reduced white matter integrity in the anterior corona radiata in major depression. J. Affect. Disord..

[62] Boato F (2013). Absence of IL-1β positively affects neurological outcome, lesion development and axonal plasticity after spinal cord injury. J. Neuroinflamm..

[63] Prins M (2013). Interleukin-1β and interleukin-1 receptor antagonist appear in grey matter additionally to white matter lesions during experimental multiple sclerosis. PLoS One.

[64] Cai Z, Lin S, Pang Y, Rhodes PG (2004). Brain injury induced by intracerebral injection of interleukin-1beta and tumor necrosis factor-alpha in the neonatal rat. Pediatr. Res..

[65] Grippo AJ, Francis J, Beltz TG, Felder RB, Johnson AK (2005). Neuroendocrine and cytokine profile of chronic mild stress-induced anhedonia. Physiol. Behav..

[66] Xu H (2015). Changes in proinflammatory cytokines and white matter in chronically stressed rats. Neuropsychiatr. Dis. Treat..

[67] Swardfager W (2017). Peripheral inflammatory markers indicate microstructural damage within periventricular white matter hyperintensities in Alzheimer’s disease: A preliminary report. Alzheimers Dement. Diagn. Assess. Dis. Monit..

[68] Chiang P-L (2017). White matter damage and systemic inflammation in Parkinson’s disease. BMC Neurosci..

[69] Baer M (1998). Tumor necrosis factor alpha transcription in macrophages is attenuated by an autocrine factor that preferentially induces NF-B p50. Mol. Cell Biol..

[70] Chen L, Zeng X, Zhou S, Gu Z, Pan J (2022). Correlation between serum high-sensitivity C-reactive protein, tumor necrosis factor-alpha, serum interleukin-6 and white matter integrity before and after the treatment of drug-naïve patients with major depressive disorder. Front. Neurosci..

[71] Magioncalda P (2018). White matter microstructure alterations correlate with terminally differentiated CD8+ effector T cell depletion in the peripheral blood in mania: Combined DTI and immunological investigation in the different phases of bipolar disorder. Brain. Behav. Immun..

[72] Doolin K, Farrell C, Tozzi L, Harkin A, Frodl T, O'Keane V (2017). Diurnal hypothalamic–pituitary–adrenal axis measures and inflammatory marker correlates in major depressive disorder. Int. J. Mol. Sci..

